# “Much ado to achieve nothing: prospects for curing HIV infection”

**DOI:** 10.1186/2052-8426-2-9

**Published:** 2014-03-26

**Authors:** Andrew D Badley

**Affiliations:** Division of Infectious Diseases, Mayo Clinic, Rochester, Minnesota USA; Division of Molecular Medicine, Mayo Clinic, Rochester, Minnesota USA

**Keywords:** HIV, Cure, Gene therapy, Reactivation, Immune based therapy

## Abstract

Currently there is significant scientific effort being directed at developing ways to create either a sterilizing cure, or functional cure for HIV infection. Multiple approaches are being evaluated under the broad headings of gene therapy, immune based interventions, and treatments which depend upon HIV reactivation from latency to cause the death of cells which harbor the virus. *Molecular and Cellular Therapies* (*MCT*) welcomes all manuscripts devoted to increasing our understanding of determinants of affecting a cure for HIV and mechanistic studies determine the cellular and viral interventions necessary for achieving HIV cure.

More than 30 years since the discovery of Human Immunodeficiency Virus (HIV), advances in our understanding of the disease, as well as, development and optimization of effective drug combinations, have together transformed HIV from an almost uniformly fatal disease to one that is manageable for long periods of time. Now for patients infected with HIV who have access to effective antiretroviral therapy, viral replication can be controlled for long periods of time and the resultant immunodeficiency prevented. However, there is considerable morbidity and mortality associated with long term antiviral suppression of HIV; notably accelerated aging, and an increased incidence of the diseases associated with aging include accelerated Cardiovascular Disease, cognitive impairment, cancer, osteoporosis, and other end-organ diseases such as liver disease, kidney disease, and bone disease [1]. Therefore, there is still a need for interventions which prevent and/or cure HIV.

There are two mutually exclusive, and possibly equally desirable forms of cure; sterilizing cure, wherein the host acquires undetectable viral nucleic acid sequences or proteins, and functional cure, wherein HIV remains detectable, however the host can spontaneously control viral replication to such a degree that immunodeficiency does not result, and antiviral therapy is not necessary. Recent years have witnessed several successes which demonstrate the proof of concept that both functional and sterilizing cure are possible. 14 French patients, in the Visconti cohort, who initiated combination anti-retroviral therapy (ART) during acute infection, were maintained on ART for several years after infection. Following ART discontinuation, this population of patients were able to spontaneously control viral replication and maintain stable CD4 counts over, as many as, 12 years of follow-up [2]. These cases demonstrate the feasibility of a functional cure for HIV, although the mechanisms by which HIV was controlled in these patients as opposed to other patients treated during acute infection that do not spontaneously control viremia remains unknown. Secondly, sterilizing cure has also been achieved. The first and best characterized case is of a gentleman with HIV infection who was controlled on long term anti-viral therapy that was diagnosed with acute myeloid leukemia (AML) and underwent allogeneic stem cell transplantation using cells from an unrelated donor who had a homozygous CCR5∆32 mutation. More than five years after his transplant, and cessation of ART, he remains free of detectable HIV [3]. A second case of sterilizing cure has occurred in a baby from Mississippi, who was born to an HIV infected mother, and consequently received ART prophylaxis beginning at 30 hours of age. HIV DNA and RNA were detected at 30 hours and 31 hours of life respectively, and RNA detected at 3 other time points within the first three weeks. At 23 months of age, the mother reported that ART had been discontinued at 18 months of age, yet plasma viremia remained negative through 30 months of age, and viral outgrowth assays performed at 24 months of age did not yield detectable HIV [4].

The challenge that remains for the HIV research community is to identify ways of generalizing the ability to cure HIV to the wider HIV infected patient community.

## Possible pathways toward a cure

### Shock and kill hypothesis

The Shock and Kill Hypothesis predicts, in essence, that because HIV proteins are intrinsically cytotoxic, reactivation of HIV from latency should result in the death of those cells induced to express HIV proteins. When reactivation occurs in a patient who has viral replication suppressed by ART, repopulation of the HIV reservoir will be prevented and ultimately HIV might be cured. Progress in the arena has been most notable in the area of developing approaches to reactivate HIV. In this regard, there are a number of described HIV reactivators with therapeutic potential, including CD3/CD28 stimulation, mitogens such as PHA, calcium ionophores, stimulation with IL-2 or IL-7, HMBA which targets P-TEFb, histone deacetylase inhibitors, and others [5]. Despite these advances, HIV reactivation from latency does not result in death of the reactivation cell [6]. Possible mechanisms that underline this lack of death include intrinsic apoptosis resistance of the cells which represent a long lived reservoir for HIV [7]. Other possibilities include an ineffective immune response against HIV such that the immune system does not eradicate those cells induced to express HIV proteins [8]. Therefore, studies which address ways of converting HIV reactivation without death, to HIV reactivation and death of the formerly latently infected cell, are of great interest.

### Vaccination and immune based therapies

There have been numerous trials of vaccination for HIV as a means to either prevent and or treat HIV infection. Largely these studies have been ineffective. Noteworthy however is the Thai Trial, which tested recombinant canarypox vector vaccine (ALVAC-HIV [vCP1521]) plus two booster injections of a recombinant glycoprotein 120 subunit vaccine (AIDSVAX B/E), as it showed approximately 30% reduction in the incidence of HIV acquisition amongst 16,402 study subjects [9]. The Thai Trial didn’t test a unique vaccination strategy per se, however, it was conducted in a unique cohort of Thai patients, which may underline its success. Indeed studies in rhesus macaques, have shown that therapeutic vaccination may be genotype specific; i.e. different HLA backgrounds will require different vaccine antigens to confirm protective immunity [10]. An alternate possibility is that this vaccine generated weakly neutralizing antibodies [11], thereby generating renewed interest in broadly neutralizing antibodies as a therapeutic or prophylactic target [12]. Other areas of promise within immune based therapies include chimeric antigen receptor approaches, and immunotoxins. Chimeric antigen receptor (CAR) expressing T cells have been in clinical trials for a variety of malignant diseases, with early promising results [13] and represent the variable light and heavy chains from immunoglobulin, fused to the TCR zeta chain and intracellular signaling domains from the costimulatory molecules CD28 with 41BB and/or OX40. T Cells modified to express CARs can persist for over 11 years after infusion [14], and in cancer cell systems, have been effective at eliminating target cells [15]. This technology is now being applied to HIV, and in vitro results suggest that CAR modified T cells can both reactivate HIV from latency, and kill the reactivating cells [16].

Another interesting and promising approach to immune based therapy for HIV cure is HIV specific immunotoxin therapy. First proposed several decades ago the approach simply, is to target those cells which express HIV proteins (typically Gp120) with synthetic proteins with a recognition domain (e.g. CD4 binding domain for gp120) and a toxin (e.g. pseudomonas Exotoxin). This approach has shown efficacy in vitro, and proceeded as far as early phase clinical trials, however since these studies were performed in an era when ART was incompletely suppressive, there was no sustained effect on viral burden [17]. Now the concept has been retested in the human bone marrow, liver thymus (BLT) mouse model, and in concert with suppressive ART, has been shown to significantly reduce HIV burden [18].

### Gene therapy approaches

The success of allotransplantation with CCR5 null donor cells has led to numerous attempts to inactivate CCR5 and other host proteins necessary for HIV replication, using either sh RNA, zinc finger nucleases (ZFN), or transcription activator like effector nucleases (TALENS), and several of these approaches have advanced to early phase clinical trials [19]. A recent open label pilot clinical trial of CCR5 deleted ZFN modified autologous CD4 T cells administered to HIV infected patients on suppressive ART demonstrates safety feasibility and biologic activity of maintaining CD4 T cell number following ART cessation [20]. Other proposed approaches include exploiting our advancing knowledge concerning the HIV restriction factors APOBEC, SAMHD1, Tetherin and TRIM5a to therapeutic benefit. Indeed, expression of modified APOBEC3G [21] and TRIM5 [22] have shown anti HIV effects in vitro. Finally genetic overexpression of HIV fusion antagonists such as mC46 may have the added benefit of protective effects on unmodified bystander cells, [23]. While these approaches are a long way from clinical applicability, there are ongoing clinical trials of these approaches in conjunction with bone marrow transplant which could yield promising insights into genetic approaches in curing HIV. Importantly, instructed by experience with ART, proponents of genetic therapies for HIV are adopting combination approaches to minimize the risk of viral escape, and several such studies are currently underway in humans (NCT 01153646, NCT00569985, NCT01734850).

### Ongoing challenges for HIV cure research

The principal barrier to a cure to HIV is the existence of long lived cells containing latent HIV, which by virtue of latency are resistant to either the cytotoxic effects of immunity, and the antiretroviral effect of combination therapy. These cells are infrequent in vivo, and therefore difficult to study using cells from HIV infected patients. Various groups have proposed model systems to recapitulation latency in vitro, however recent research has indicated that these model systems have variable phenotype that act variable to different reactivation stimuli, and therefore may not truly reflect the in vivo situation [24]. Thus, more predictive models of HIV latency are sorely needed. In addition, sensitive detection of the viral reservoir is notoriously difficult, owing to the very low level HIV DNA contained within latently infected cells. A variety of approaches have been proposed to measure this latent reservoir including Alu nested PCR, Total Cell Associated HIV DNA, and quantitative viral out growth assays, however there is still need for improvement, and enhanced means of detecting ultra-low level HIV. Finally, model animal systems that permit the study and treatment of HIV latency and persistence present an ongoing challenge. The recent development of the human bone marrow, liver thymus mouse model, represents an advance, however this model is cumbersome expensive and not widely available, and therefore more research is needed.

*MCT* encourages and welcomes original scientific research, reviews, commentaries, methodologies, short reports and systematic reviews related to the issues discussed in this editorial.
